# Near-Infrared Spectroscopy for Neonatal Sleep Classification

**DOI:** 10.3390/s24217004

**Published:** 2024-10-31

**Authors:** Naser Hakimi, Emad Arasteh, Maren Zahn, Jörn M. Horschig, Willy N. J. M. Colier, Jeroen Dudink, Thomas Alderliesten

**Affiliations:** 1 Department of Neonatology, Wilhelmina Children’s Hospital, University Medical Center Utrecht, Lundlaan 6, 3584 EA Utrecht, The Netherlands; nh.naserhakimi@gmail.com (N.H.); e.arastehemamzadehhashemi@umcutrecht.nl (E.A.); j.dudink@umcutrecht.nl (J.D.); 2 Donders Institute for Brain, Cognition and Behaviour, Radboud University, P.O. Box 9103, 6500 HD Nijmegen, The Netherlands; marenzahn@gmail.com; 3 Artinis Medical Systems, B.V., Einsteinweg 17, 6662 PW Elst, The Netherlands; jorn@artinis.com (J.M.H.); willy@artinis.com (W.N.J.M.C.)

**Keywords:** near-infrared spectroscopy, neonatal sleep, sleep classification, brain monitoring, heart rate, respiratory rate

## Abstract

Sleep, notably active sleep (AS) and quiet sleep (QS), plays a pivotal role in the brain development and gradual maturation of (pre) term infants. Monitoring their sleep patterns is imperative, as it can serve as a tool in promoting neurological maturation and well-being, particularly important in preterm infants who are at an increased risk of immature brain development. An accurate classification of neonatal sleep states can contribute to optimizing treatments for high-risk infants, with respiratory rate (RR) and heart rate (HR) serving as key components in sleep assessment systems for neonates. Recent studies have demonstrated the feasibility of extracting both RR and HR using near-infrared spectroscopy (NIRS) in neonates. This study introduces a comprehensive sleep classification approach leveraging high-frequency NIRS signals recorded at a sampling rate of 100 Hz from a cohort of nine preterm infants admitted to a neonatal intensive care unit. Eight distinct features were extracted from the raw NIRS signals, including HR, RR, motion-related parameters, and proxies for neural activity. These features served as inputs for a deep convolutional neural network (CNN) model designed for the classification of AS and QS sleep states. The performance of the proposed CNN model was evaluated using two cross-validation approaches: ten-fold cross-validation of data pooling and five-fold cross-validation, where each fold contains two independently recorded NIRS data. The accuracy, balanced accuracy, F1-score, Kappa, and AUC-ROC (Area Under the Curve of the Receiver Operating Characteristic) were employed to assess the classifier performance. In addition, comparative analyses against six benchmark classifiers, comprising K-Nearest Neighbors, Naive Bayes, Support Vector Machines, Random Forest (RF), AdaBoost, and XGBoost (XGB), were conducted. Our results reveal the CNN model’s superior performance, achieving an average accuracy of 88%, a balanced accuracy of 94%, an F1-score of 91%, Kappa of 95%, and an AUC-ROC of 96% in data pooling cross-validation. Furthermore, in both cross-validation methods, RF and XGB demonstrated accuracy levels closely comparable to the CNN classifier. These findings underscore the feasibility of leveraging high-frequency NIRS data, coupled with NIRS-based HR and RR extraction, for assessing sleep states in neonates, even in an intensive care setting. The user-friendliness, portability, and reduced sensor complexity of the approach suggest its potential applications in various less-demanding settings. This research thus presents a promising avenue for advancing neonatal sleep assessment and its implications for infant health and development.

## 1. Introduction

Infants born prematurely spend their early weeks in an incubator instead of the protective environment of the mother’s womb [1]. This period is crucial for their brain development because the latter part of the second semester and the third trimester are critical for creating new connections in the brain [2]. Fetal sleep, which is believed to be the primary driver of neural activity, is vital for neuronal survival, axonal guidance, and synapse maturation [3,4]. However, infants in the neonatal intensive care unit (NICU) receive hands-on care and might be exposed to various external stimuli that can significantly disrupt their sleep [5].

The significance of sleep for neonatal brain development is being increasingly acknowledged [6,7]. Disrupted sleep during the neonatal period has already proved to have negative developmental consequences [8]. Monitoring neonatal sleep continuously in the NICU could aid care in two ways. First, as neonatal sleep is considered to reflect typical brain maturation, it can also serve as a biomarker for future outcomes, such as the severity of an illness or the infant’s maturational state [9]. Second, if sleep is monitored in real-time, elective care could be individually customized to sleep states to ensure optimal brain development [10,11].

The primary method of assessing sleep in very preterm infants is behavioral observation [12]. Sleep comprises three behavioral states: AS, which is important for creating new brain connections; QS, which is critical for consolidating the connections and recovery; and intermediate sleep, which is a transitional state [13]. Ideally, elective care should correspond with neonatal sleep states, but this requires continuous sleep observation by trained observers, which is time-consuming and costly and therefore not feasible [14]. However, sleep states in (pre) term infants can also be determined based on typical HR and RR patterns [15]. Besides, video-based methods are gaining popularity for non-invasive and contactless classification of sleep and wake states in neonates. The unobtrusive and easy-to-use nature of video-based methods makes them suitable for long-term monitoring in both home and clinical settings [16].

Numerous studies have delved into the utilization of machine learning and neural network classification techniques to automatically detect sleep–wake states in infants. Sentner et al. [17] and Werth et al. [14] integrated physiological characteristics such as HR, RR, and peripheral oxygen saturation (SpO2) from regular monitor recordings in the NICU to classify sleep states in preterm infants. Other studies utilized electroencephalography (EEG) features for sleep classification. Specifically, Hermans et al. [18] developed a CNN classifier that fused multiple EEG-derived features to differentiate between sleep states in term infants. Besides, Wang et al. [19] exclusively utilized EEG features in preterm infants. Koolen et al. [20] proposed a Support Vector Machines (SVM) classifier trained on EEG features in infants, and Ansari et al. [21] used exclusively EEG features to classify QS vs. non-QS in preterm infants. These studies demonstrate the potential of machine learning and neural network classification techniques in accurately identifying sleep states in neonates.

Despite the promising results of the automated sleep-staging methods, they have some limitations that make them difficult to use for continuous daily monitoring in the NICUs. For instance, some methods need multiple electrodes that can potentially damage the delicate skin of preterm infants [20,21,22]. Additionally, most of the existing studies, including those mentioned above, have only classified sleep states using pooled datasets, which lacks subject-specific analysis of preterm infants’ sleep.

NIRS is a non-invasive technique that utilizes varying wavelengths of infrared light to monitor changes in oxygenated and deoxygenated hemoglobin in tissues, with venous circulation contributing significantly to the signal [23]. Therefore, the effects of many physiological signals of the human body are embedded in recorded NIRS signals [24]. In this regard, recently, NIRS has demonstrated its ability to capture various physiological parameters such as heartbeats, respiration, and blood pressure [23,24,25].

HR and RR have been accurately derived from high sampling rate NIRS signals in both adults and neonates [26,27,28]. Extracting HR and RR from NIRS not only allows for the measurement of diverse physiological information using a single sensor but also opens avenues for exploring multi-modal feature extraction to assess sleep states, particularly in infants, a domain yet unexplored. This innovative approach holds the potential to offer a non-invasive and simpler alternative to traditional polysomnography (PSG), minimizing discomfort for preterm infants by employing only a single small head-mounted sensor.

This study introduces a new method for assessing sleep in preterm infants recorded in a complex hospitalized environment. We leverage the capabilities of high sampling rate NIRS to extract various features, including heart rate, respiratory rate, motion-related indicators, and neuronal activity. The single-channel NIRS allows for instantaneous time-synced multimodal data analysis from a single sensor, offering an efficient bedside monitoring system that minimizes discomfort compared to the multichannel EEG routinely used for sleep assessment. These features are then analyzed using a CNN-based classifier to distinguish between AS and QS states. Our study not only showcases the potential of high sampling rate NIRS for detailed sleep analysis but also emphasizes the practicality of our approach in clinical settings, utilizing a single sensor. This innovative approach enhances neonatal sleep state assessment, potentially providing a simplified and less intrusive alternative to traditional PSG, reducing discomfort for preterm infants.

## 2. Materials and Methods

### 2.1. Participants

This study was carried out within the NICU at the Wilhelmina Children’s Hospital, Utrecht, the Netherlands. The inclusion criterion was a gestational age (GA) of less than 37 weeks. Exclusion criteria were genetic anomalies, congenital malformations, historical occurrences of seizures, evident brain injuries (e.g., intraventricular hemorrhage exceeding grade 2 according to the classification by Papile [29]), and maternal utilization of recreational drugs during pregnancy. The study involved 13 infants, resulting in 17 measurements, with 4 infants contributing 2 measurements each. However, we excluded data from four infants due to factors such as recent administration of sedatives within 24 h before recording, sensor malfunction, or the absence of reference data. Consequently, our analysis was performed on a dataset comprising 10 measurements obtained from nine infants. Prior to participation in the study, written parental informed consent was obtained. This research was approved by the local Medical Ethical Review Committee (METC number 21-098/C). Clinical characteristics and included data for each subject individually are presented in Table 1.

### 2.2. Data Acquisition

**Figure 1:**
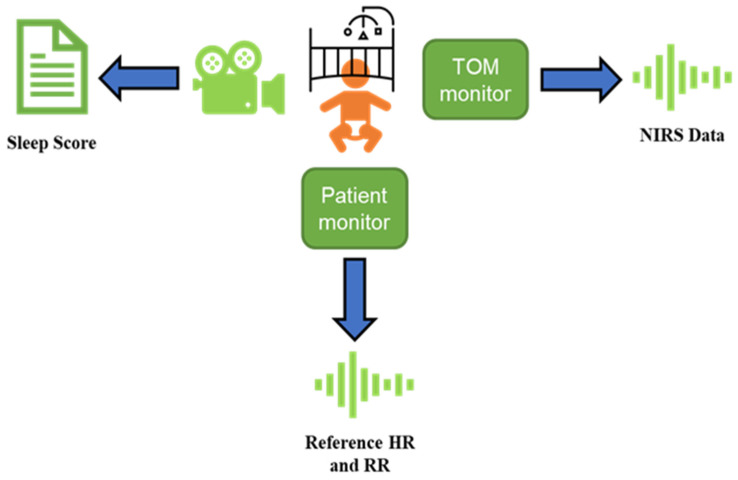
Schematic representation of the data acquisition protocol in this study.

A schematic overview of the data acquisition procedure is presented in Figure 1. NIRS data, encompassing measurements of oxygenated hemoglobin (O2Hb), deoxygenated hemoglobin (HHb), and tissue saturation index (TSI), were acquired using the TOM device (Artinis Medical Systems B.V., Elst, The Netherlands) at a sampling rate of 100 Hz. The TOM sensor, equipped with two NIRS channels, was positioned on the neonate’s forehead, precisely above and parallel to the eyebrows. The sensor was secured beneath a CPAP (continuous positive airway pressure) cap or headband (elastic bandage) made of biocompatible materials to minimize the risk of skin irritation or discomfort. The cap or headband was lightweight and designed to remain stable during monitoring without restricting the infant’s movement or causing excessive pressure. Reference HR and RR values were obtained using a bedside Philips IntelliVue MP70 (Philips Medical Systems, Best, The Netherlands) stored at 0.4 Hz. These reference data were collected to help evaluate the accuracy of the algorithms employed for HR and RR extraction from the NIRS data. Throughout the entire recording period, the facial and bodily expressions of the infants were concurrently captured by one or two cameras. These cameras, specifically the Intel^®^ RealSense Depth Camera D435i (Intel Corporation, Santa Clara, CA, USA) and the 1SEE VDO360 camera (VDO360, Edgewater, MD, USA), provided a frame rate of 30 frames per second and a resolution of 1920 × 1080 pixels. Data synchronization was achieved in a two-step process. First, the time settings on each device were synchronized with internet time. Second, both the internet time and the device’s individual measurement start time were adjusted to achieve accurate synchronization of the collected data.

### 2.3. Sleep Observation

A validated behavioral sleep state scoring system, known as the “Behavioral Sleep Stage Classification for Preterm Infants (BeSSPI)”, recommended as the gold standard for this age group, was employed for sleep state classification. In preterm infants, conventional EEG-based sleep staging rules are not applicable. For more details, refer to [12]. A trained annotator (MZ) used video data to categorize sleep states, with a specific focus on two primary states: AS and QS. These classifications were performed at one-minute intervals. We used 1-min epochs as recommended in the BeSSPI score, long enough to observe all parameters multiple times but short enough to prevent a transition to a different sleep state during the epoch. There are two advantages of this scoring method compared to scoring systems using electrophysiological data. First, placing electrodes can be a burden on (preterm) infants and restricts the possibility of conducting sleep research outside the NICU [30]. Secondly, EEG-based scoring methods have already been shown to be prone to mistakes for preterm infants with GA < 30 weeks [31]. The final annotation includes 191 (QS) vs. 570 (non-QS; 38 Wake and 532 AS) 1-min sleep scores.

### 2.4. NIRS Data Analysis

The concentration changes in O2Hb and HHb were determined using the modified Beer–Lambert law [32]. The Beer–Lambert law relates light attenuation to the properties of the tissue through which the light travels and can be expressed in the following form:

(1) $$A=\in .l.c=-\log\frac{I}{I_{0}}$$

where A, ∈, l, c, I, and I_0_ are the absorbance, molar extinction coefficient, optical path length, concentration of the chromophores, intensity of light received by the receiver, and transmitted through the tissue, respectively. The modified Beer–Lambert law extends the original equation to account for light scattering in biological tissues and increased distance the light travels:

(2) $$OD=-\log\frac{I}{I_{0}}=\in .\Delta c.DPF.l+G$$

where OD, DPF, and G represent the optical density, differential path length factor, and scattering loss, respectively. This formulation indicates that variations in light attenuation are proportional to the changes in tissue chromophore concentrations. By measuring changes in attenuation or optical density at two or more wavelengths, the concentration changes of O2Hb and HHb can be determined. For a more detailed explanation of extracting O2Hb and HHb signals from NIRS optical densities, refer to the work by Delpy et al. [32].

Subsequently, the Signal Quality Index (SQI) algorithm [33] was employed to assess the signal quality of each NIRS channel. SQI algorithm quantitatively assesses NIRS signal quality on a 1-to-5 scale. It employs two preprocessing steps—converting raw light intensities to optical densities and applying a band-pass filter—before three rating stages: detecting very low-quality signals, identifying very high-quality signals, and rating intermediate signals. For a more comprehensive explanation of the SQI algorithm, refer to Sappia and Hakimi et al. [33]. The channel with the highest signal quality was selected for further analysis.

To extract HR and RR from the NIRS signals, we utilized the algorithms developed by Hakimi et al. [27,28]. This involved segmenting the total hemoglobin concentration (tHb) signal, which is the addition of O2Hb and HHb signals, and the normalized interquartile range (IQR) signal using sliding windows of 30 s with a 75% overlap (i.e., shifted 7.5 s). Subsequently, we computed the spectrum of the converted tHb signal and identified the dominant frequencies within the adaptive HR and RR frequency bandwidths. These dominant frequencies represented the extracted HR and RR values derived from the NIRS signals, respectively. The performance of the algorithms for extracting HR and RR was assessed using various metrics, including Mean Error (ME), Root Mean Square Error (RMSE), Pearson’s correlation coefficient, and Bland–Altman Limits of Agreement (LoA), providing a comprehensive evaluation of their accuracy and effectiveness.

### 2.5. Sleep Classification

#### 2.5.1. Multi-Modal Feature Extraction

We extracted eight features, treated as modalities, as time series data sampled at 1 Hz from the NIRS signals. By the methodology detailed in Section 2.4, the extraction of HR and RR from the NIRS signals occurred at 7.5-s intervals. Subsequently, we applied spline interpolation to convert the extracted HR and RR values into time series with a 1 Hz sampling rate.

For features related to motion artifacts present in the NIRS signals [34,35,36], we calculated the standard deviation and IQR based on the O2Hb signal within sliding windows of 1-s duration, with a 50% overlap. These computed values were then subjected to spline interpolation to achieve a 1 Hz sampling rate. To normalize these features, we divided the standard deviation by the signal’s mean and the IQR by the signal’s median. Additionally, we computed the Pearson’s correlation between the O2Hb and HHb signals within the same sliding window and interpolation procedure. This feature serves a dual purpose [37,38], potentially indicating brain activation when exhibiting a strong negative correlation or, conversely, motion artifacts when demonstrating a strong positive correlation. Furthermore, for the remaining features, we applied a 1-s moving average filter to the O2Hb, HHb, and TSI signals. Subsequently, these signals were down-sampled to 1 Hz.

Table 2 provides a comprehensive summary of the eight features derived from the NIRS signals. Notably, since the sleep scores were assigned in 1-min intervals and considering the 1 Hz sampling rate, each modality consisted of 60 data samples for analysis.

#### 2.5.2. Classification

**Figure 2:**
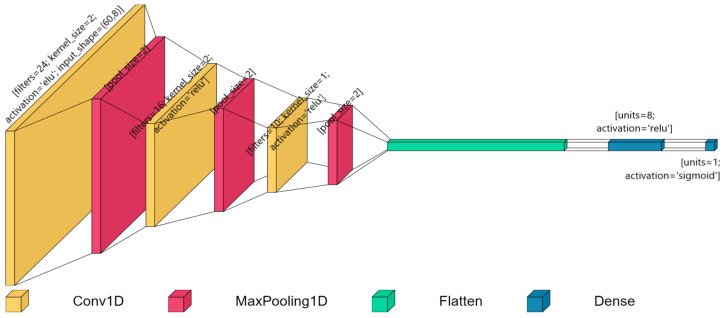
Graphical representation of the CNN model proposed in this study.

We employed six benchmark classifiers: K-Nearest Neighbors (KNN), Naive Bayes (NB), SVM, Random Forest (RF), AdaBoost (AdaB), and XGBoost (XGB) [39], alongside a novel classifier based on CNN. A visual depiction of the proposed CNN model can be seen in Figure 2.

The classifiers underwent training and testing utilizing the features extracted as described in Section 2.5.1, employing two distinct approaches. Initially, a data pooling approach was executed, where all measurements were consolidated, and the classifier was trained and tested through 10-fold cross-validation on the merged dataset. In the training phase, class weights (only of training data) were employed using the inverse frequency of each class to address class imbalance by assigning higher weights to less frequent classes. In imbalanced classification problems, errors in the minority class are deemed more critical, necessitating performance metrics focused on the minority class despite the challenge of limited observations for effective model training [40]. Therefore, in this study, for computation of the confusion matrix, QS is considered the positive class based on information in Table 1, which shows it as the minority class. Subsequently, a 5-fold cross-validation approach (each fold including two independent NIRS recordings) was adopted to ensure the model’s generalizability across individual subject-specific variations. We called it here leave-measurement-out cross-validation because two of the measurements are from the same subject, and that is our approach’s difference from leave-subject-out cross-validation. In this approach, the 10 measurements (from 9 subjects) were partitioned into 5 groups (referred to as Group 1 through Group 5), ensuring that each subject was exclusively represented in one group and minimizing the difference in the ratio of AS to QS events between the 5 groups. Consequently, the classifier underwent training on data from four groups while its performance was assessed for the remaining group in a repeatable loop. Table 3 provides a summary of the optimized hyperparameters for the benchmark classifiers, determined through hyperparameter optimization utilizing the GridSearchCV package in Python [41]. Ranges of hyperparameter values that were evaluated are listed in Supplementary Table S1. Classifier performance was assessed through metrics like accuracy, balanced accuracy, F1-score, Kappa, and AUC-ROC analysis. To assess the sensitivity of the trained model to the age of the subjects, we have also computed the Pearson’s correlation between the performance of the model (based on leave-one-out subject cross-validation) for subjects and their PMA. Training data are bootstrapped 10 times to form cross-validation of the training set with bootstrapping (resampling with replacement) to have a more trustable sensitivity analysis [42]. The learning algorithm included Adam optimizer, binary-cross entropy loss function, learning rate of 0.001, and batch size of 2.

## 3. Results

### 3.1. Results of HR and RR Extraction

**Figure 3:**
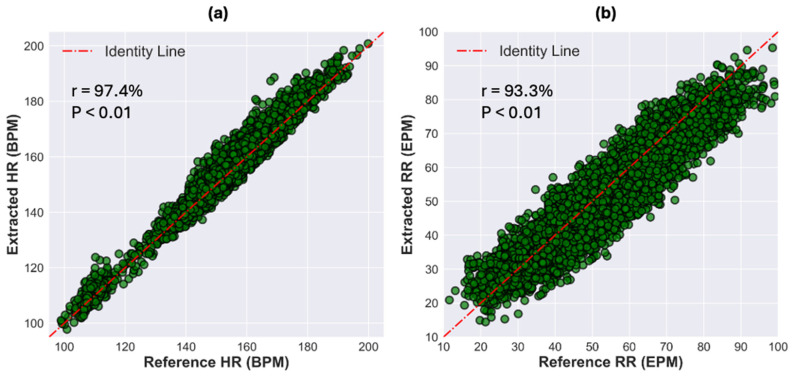
(**a**) Scatter plot between the reference HR and the extracted HR from NIRS when all measurements are concatenated (in BPM, which stands for beats per minute). (**b**) scatter plot between the reference RR and extracted RR from NIRS when all measurements are concatenated together (in EPM, which stands for events (i.e., breaths) per minute). Each green dot corresponds to a 30-s signal segment.

Figure 3 depicts scatter plots showcasing the relationship between the extracted HR from NIRS and the reference HR in Figure 3a, and similarly, the extracted RR from NIRS in Figure 3b against the reference RR. Note that HR and RR were measured and computed for units of beats per minute (BPM) and breaths per minute (referred to as EPM, events per minute), respectively. Notably, both scatter plots reveal a statistically significant (*p* < 0.01) linear correlation between the extracted physiological metrics (HR and RR) and their corresponding reference values. Specifically, the Pearson’s correlation analysis reveals a strong correlation of 97.4% for HR and 93.3% for RR between the reference and extracted data. Looking at the scatter plots, we observe that the algorithms utilized for HR and RR extraction from NIRS effectively estimate the reference physiological parameters across a range of values. Table 4 summarizes the quantitative metrics averaged on all measurements used for validating the algorithm’s performance, extracting HR and RR from NIRS. For HR extraction, the ME and RMSE between the reference and extracted HRs were computed at −1.4 ± 0.2 BPM and 3.4 ± 0.2 BPM, respectively. In the case of RR extraction, the ME and RMSE were slightly higher in magnitude, calculated at −2.1 ± 0.5 EPM and 6.1 ± 0.2 EPM, respectively. Additionally, the LoA for HR extraction was narrower, at 6.0 ± 0.3 BPM compared to RR extraction, where it was 11.1 ± 0.5 EPM. Furthermore, Pearson’s correlation analysis yielded a correlation of 93.9 ± 4.2% (*p* < 0.01) for HR extraction and 89.3 ± 6.6% (*p* < 0.01) for RR extraction, reaffirming the robustness of the algorithm in accurately estimating the reference physiological information.

### 3.2. Results of Sleep Classification with the Data Pooling Cross-Validation Approach

Table 5 summarizes numerically the average and standard deviation values for each quantitative metric across all measurements. Employing the data pooling cross-validation approach, our proposed CNN classifier consistently outperforms benchmark classifiers, yielding an average accuracy of 88%, balanced accuracy of 94%, F1-score of 91%, Kappa of 95%, and AUC-ROC of 96%. In Supplemental Figure S1, the average value of each feature in the pooled dataset (i.e., 723 epochs) and the corresponding label (i.e., AS or QS) are plotted

In contrast, benchmark classifiers, such as SVM and NB, consistently demonstrate performance levels lower than the best-performing classifiers. The AdaB and KNN classifiers, while not reaching the highest levels of performance, also exhibit notable performance, while RF and XGB classifiers excel in terms of accuracy, averaging 88% and 86%; Kappa, 65% and 62%; and AUC-ROC, 85% and 87%, respectively. However, it is important to note that their superior accuracy comes at the expense of performance across other metrics, which remains notably lower than that of the proposed CNN classifier.

### 3.3. Results of Sleep Classification with Leave-Measurement-Out Cross-Validation Approach

The leave-measurement-out cross-validation approach includes the data of two measurements as a test set at each of the five folds. Based on the subject numbers mentioned in Table 1, the test subjects’ numbers in the five folds are: {2,9} (total duration of 78 min), {4 (first trial), 4 (second trial)} (total duration of 112 min), {1,3} (total duration of 149 min), {7,8} (total duration of 131 min), and {5,6} (total duration of 253 min).

Table 6 is a summary of the average and standard deviation values for each quantitative metric across all measurements, employing the leave-measurement-out cross-validation approach. Regarding accuracy, the proposed CNN, RF, and XGB classifiers demonstrated comparable performance, achieving the highest average accuracy and AUC-ROC among all classifiers at 78% and approximately 75%, respectively. The proposed CNN classifier showcased superior performance in terms of balanced accuracy (72 ± 6%), F1-score (59 ± 10%), and Kappa (44 ± 14%). Among the benchmark classifiers, the NB classifier recorded the highest values for balanced accuracy (70 ± 8%) and F1-score (55 ± 6%), albeit with lower accuracy (68 ± 11%). The XGB classifier exhibited the highest Kappa (38 ± 21%), although it displayed a relatively higher standard deviation.

Table 7 summarizes numerically the accuracy, balanced accuracy, F1-score, Kappa, and AUC-ROC for the proposed CNN classifier per group when using the leave-measurement-out cross-validation approach. Among the subject groups, the classifier evaluated in Group 1 (G1) demonstrated the highest levels of performance across multiple metrics, including accuracy (86%), balanced accuracy (81%), F1-score (74%), Kappa (65%), and AUC-ROC (88%). In contrast, the classifier assessed in Group 3 (G3) displayed the lowest performance, recording accuracy (70%), balanced accuracy (63%), F1-score (48%), Kappa (27%), and AUC-ROC (72%) at the lowest levels. The most pronounced variation among the groups was observed in the Kappa performance metric with a range of 38%, while the smallest degree of variation was found in the accuracy and AUC-ROC metrics with a range of 16%.

The correlations between PMA and accuracy, balanced accuracy, F1-score, and Kappa are (r = 1%, *p*-value = 0.8), (r = 21%, *p*-value = 0.1), (r = 19%, *p*-value = 0.1), and (r = 14%, *p*-value = 0.21), respectively. Therefore, there is no direct relation between the performance of the sleep assessment by the CNN model and the PMA of the subjects.

## 4. Discussion

In this study, we explored the use of a multi-modal feature extraction algorithm applied to a high sampling rate NIRS signal (acquired by a single sensor) to assess its potential for characterizing sleep states in preterm infants. Utilizing a CNN classifier, we differentiated AS from QS by extracting and combining various features from the high sampling rate NIRS, including physiological signals such as HR, RR, motion-related information, and raw NIRS signals. Our approach allowed for the assessment of NIRS signal quality and the extraction of diverse physiological and non-physiological features, including motion artifacts and brain activation, providing a comprehensive platform for sleep assessment. By combining these features, which have not been widely used in previous studies, we created a method that shows potential for assessing sleep patterns in preterm infants using data from a single sensor.

This classifier, distinguishing between AS that aids neural processing and learning consolidation [43] and QS that promotes physical growth and recovery [44,45], applied on data with minimal contact to the preterm, can be of pivotal benefit to both clinical and research settings.

The performance of the proposed classifier was evaluated through a quantitative analysis involving key performance metrics, including accuracy, balanced accuracy, F1-score, Kappa, and AUC-ROC. The assessment of classifier performance was undertaken via two distinct cross-validation strategies, namely, pooling cross-validation and leave-measurement-out cross-validation. In the context of pooling cross-validation employing a 10-fold scheme, the proposed classifier achieved an accuracy of 88 ± 12%, a balanced accuracy of 94 ± 7%, an F1-score of 91 ± 9, a Kappa of 95 ± 5%, and an AUC-ROC of 96 ± 4%. Moreover, we also assessed the outcomes of the classifier within the leave-measurement-out cross-validation paradigm, with a reported accuracy of 78 ± 6%, a balanced accuracy of 72 ± 6, an F1-score of 59 ± 10, a Kappa of 44 ± 14, and an AUC-ROC of 75 ± 6% (as summarized in Table 5, Table 6 and Table 7).

Furthermore, a comparative analysis was conducted to benchmark the performance of the proposed CNN classifier against six established classifiers, including SVM, AdaB, KNN, NB, RF, and XGB. Across both cross-validation methodologies, the CNN classifier consistently exhibited superior performance in terms of balanced accuracy, F1-score, and Kappa, although it is worth noting that the RF and XGB classifiers displayed competitive results in terms of overall accuracy and AUC-ROC, as detailed in Table 5 and Table 6. Notably, when considering metrics sensitive to imbalanced classification in the pooling cross-validation framework (i.e., balanced accuracy, F1-score, and Kappa), the CNN classifier demonstrated a substantial advantage over the benchmark classifiers, exceeding their performance by more than 10%. In contrast, the margin of superiority was somewhat reduced in the leave-measurement-out cross-validation approach, where the CNN classifier outperformed the benchmark classifiers by 2%, 4%, and 6% in terms of balanced accuracy, F1-score, and Kappa, respectively.

Comparing our results with other studies, we found that our method performs as well as or better than some of the existing methods, using only one sensor instead of multiple sensors. For instance, Sentner et al. [17] integrated physiological parameters such as HR, RR, and SpO2 derived from PSG recordings to delineate sleep states among 39 preterm infants, achieving an AUC-ROC ranging from 0.61 to 0.78. Similarly, Werth et al. [14] leveraged features extracted from electrocardiography to attain a Kappa score of 0.43 in the classification of AS versus QS within a cohort of 34 preterm infants. Several studies exclusively concentrated on EEG and combined diverse EEG-derived features for sleep state classification. For instance, Hermans et al. [18] devised a CNN classifier that amalgamated multiple EEG-derived features to distinguish between sleep states, achieving a Kappa score spanning from 0.33 to 0.44 among fifteen term infants. Similarly, Wang et al. [19] exclusively utilized EEG features, achieving an AUC-ROC of 0.75 in their study involving 17 preterm infants. Koolen et al. [20] introduced a SVM classifier trained on EEG features, demonstrating an accuracy and balanced accuracy of 85% across a cohort of 67 infants, while Ansari et al. [21] reported the average accuracy, balanced accuracy, and AUC-ROC values of 79%, 79.5%, and 87%, respectively, in their utilization of exclusive EEG features to discriminate between QS and non-QS states among 97 preterm infants. Comparing these outcomes with the results of our study, we conclude that it either outperforms or shows comparable results to existing methods (with the clear advantage of using only one single sensor compared to the majority of research on multi-sensor data).

It is essential to acknowledge several limitations of the current study, which warrant attention in future research. First, the extraction of HR and RR in this study relied upon algorithms outlined in [27,28] with measurements obtained at 7.5-s intervals. Notably, if sleep states exert an influence on the short-term dynamics of HR and RR, the 7.5-s interval for computation could potentially lead to the omission of critical sleep-related information, thereby potentially impinging upon the classifier’s efficacy. Nevertheless, the promising results obtained in this study confirm that the computation times for HR and RR have been appropriate for achieving precise sleep classification (AS vs. QS) in the population studied. Second, despite our assessment involving 723 one-minute (AS and QS) trials derived from a cohort of 9 subjects in a highly challenging intensive care setting, validation in a larger and more diverse patient population needs to be performed. Third, one limitation of video-based sleep scoring based on behavioral data is the subjectivity of the annotator. Therefore, the ground truth of our classification is prone to some errors and inconsistency with the main behavioral scheme developed by [12]. However, the annotators were trained by the original team that created the validated behavioral sleep score, which had a high interrater agreement. Moreover, the current research was centered on offline feature extraction and classification procedures. However, for practical clinical implementation, an online bedside version for feature extraction and classification is needed. This crucial step would serve as a litmus test to evaluate the applicability and performance of our proposed sleep classification approach within a genuine clinical environment. To progress in this direction, a key focus would involve refining the existing RR and HR algorithms to adapt them for real-time, online processing. By incorporating the preprocessing and feature extraction procedures outlined in this paper into the online system, a foundation for accurate data analysis can be established. Once the online algorithms are in place, integrating the trained classifier into this setup and then developing practical software would provide clinicians with a powerful tool for real-time sleep pattern analysis in clinical environments.

In addition, we did not use any explainable AI (XAI) method to explain each feature’s importance, as these methods can provide a descriptive representation of each feature’s weight in the learning process [46]. However, with limited data, even in the presence of good classification results, the learned representations may be noisy or biased, leading to explanations that are unreliable or difficult to interpret meaningfully [47,48]. Therefore, at this stage and regardless of proper classification results, we just proved the efficiency of the model in predicting the sleep states, and further explaining of the model needs bigger datasets to be trustworthy.

Furthermore, another limitation of this study is the binary AS vs. QS classification, despite our sleep scoring system being able to label wake and transitional sleep states. This is not a major issue in the classification of preterm infants’ sleep, as Georgoulas et al. found that the mean proportion of time spent awake is around just 5% in preterm infants during the early postnatal days. The intermediate sleep state also covers less than 5% of the early postnatal lifetime for preterm infants. Therefore, the binary classification covers about 90% of the sleep cycles for the subjects. In addition, the majority of newborns’ sleep time duration is divided between AS and QS states [49]. Therefore, the categorized predictions of our model cover the majority of during sleep QS vs. non-QS classification. This QS vs. non-QS distinction has already been shown to be beneficial in distinguishing a sleep state trend, which can be used as a potential biomarker of brain development [50]. In addition, one limitation of this research is the variation of the recorded signals’ time durations among the subjects. While there are cases of recorded (high quality) NIRS for more than 1 h, there is also a subject with 21 min of time duration. This will limit the dependability of accuracy mentioned for the subjects.

Finally, the model’s performance in this pilot study was up to ~20% for all the metrics in the case of the pooling strategy compared to the leave-measurement-out cross-validation. This is not unexpected due to the large variation in the ratio of AS to QS among subjects (Table 1). Small data samples might not completely reflect the true data distribution, potentially causing non-independent and identically distributed (i.i.d.) distributions [51]. Moreover, the outcomes of the leave-measurement (or subject)-out cross-validation approach degrade more if the datasets do not have an i.i.d. distribution [52]. In this regard, our sleep dataset has variable AS/QS ratios among subjects, and the leave-measurement-out cross-validation approach could not have reached similar outcomes like the pooling technique with this non-i.i.d. distribution of sleep states. For future studies, we aim to have a bigger dataset to make the leave-measurement (or subject)-out cross-validation a more feasible approach to be applied to the sleep classification by the NIRS data.

Current methods for monitoring sleep in preterm infants, such as PSG, while effective, have several limitations. A typical PSG setup involves multiple adhesive electrodes and sensors, including EEG electrodes on the scalp, electro-oculogram, electro-myogram on the chin and legs, and electrocardiogram electrodes [53]. Additionally, respiratory monitoring is performed using plethysmography belts, and airflow is measured with a pressure transducer and thermistor. This extensive setup, often requiring 10–15 contact points, can cause skin irritation or damage to the fragile skin of preterm infants, increasing the risk of infection [12,54]. In addition, the number of contact points is impractical as preterm infants have a very small body surface area to place the various sensors, thereby limiting continuous application of a PSG setup for monitoring. Moreover, the wires and sensors used in PSG prevent skin-to-skin contact between the infant and parent, which is crucial for bonding and the infant’s overall well-being [55].

It is also important to note that in preterm infants, the EEG component of PSG does not display the same characteristic sleep stage markers seen in older infants and adults, limiting its effectiveness as a gold standard for sleep assessment in this population. Due to these limitations, we used a validated observational scoring system (BeSSPI) tailored specifically for preterm infants as our reference method, which we believe provides a more appropriate approach for assessing sleep stages in preterm infants. While the BeSSPI provides valuable insights, it requires expert interpretation on the bedside or through video recording, limiting its practical use for real-time and prolonged monitoring.

Our proposed NIRS-based approach fills this gap by offering continuous monitoring with a single small sensor (approximately 1 × 5 cm), enabling real-time assessment of sleep states with minimal intrusion. This makes NIRS a less intrusive and more comfortable alternative to PSG. Additionally, with its continuous sleep monitoring capabilities and lack of dependence on expert interpretation, NIRS presents a more efficient alternative to the BeSSPI method. However, it is important to note that this study is a pilot investigation, and while the initial findings are promising, further validation in larger cohorts is necessary before NIRS can replace PSG or the BeSSPI as the standard of care.

There are several potential applications across diverse settings for NIRS-based sleep assessment. Foremost, it could serve as a continuous sleep monitoring tool for preterm infants in hospital settings. The simplicity of using a single head-mounted sensor in our portable system with the additional advantage of reducing potential discomfort and distress could facilitate the planning of elective care and thereby improve sleep quality. Furthermore, NIRS-based sleep assessment could be used for more precise and comprehensive home-based sleep state classification [56,57,58]. Given the increasing popularity of home-based sleep monitoring while considering the limitations of current sensor modalities [59], our NIRS-based method offers a potent alternative. Sleep analysis within the comfort of one’s own home holds great advantages, not only in terms of logistics (transportation to and from the hospital) but also in the assessment of sleep in a patient’s daily environment. This could facilitate proactive health management [60,61]. In addition, the portability and user-friendliness would enable sleep assessment in environments with limited (monitoring) resources, which could significantly augment the quality of care in such environments. Lastly, our approach introduces exciting prospects for prospective research studies in the domain of neurodevelopmental assessment, complemented by concurrent sleep monitoring [62,63]. This innovative approach holds the potential for shedding light on the intricate relationship between sleep patterns and neurodevelopmental outcomes, thus enriching our understanding of this critical aspect of neonatal health and (brain) development.

## 5. Conclusions

Our study demonstrates the viability of accurately categorizing AS and QS in preterm infants within an intensive care setting using a single high sampling rate NIRS sensor, a sophisticated feature extraction approach, and a CNN classifier. Our approach consistently outperforms in terms of balanced accuracy, F1-score, and Kappa when compared to the benchmark classifiers. Our findings offer a simplified, non-invasive, and user-friendly alternative to traditional PSG, reducing discomfort and facilitating more direct integration in daily (clinical) care.

## Figures and Tables

**Table 1:** Subject-specific clinical characteristics and data of patients included in this study.

Sub No.	GA (Weeks)	PMA (Weeks)	5-Min Apgar Score	10-Min Apgar Score	Sex	Birthweight [Grams]	Recording Duration [Minutes]	QS/AS Epoch Ratio
1	25.7	34.8	7	8	F	635	75	0.21
2	31.7	31.8	9	10	M	1355	57	0.12
3	34.6	35.1	8	9	M	1700	74	0.68
4 *	37	38.1 and 38.2	9	10	M	2650	99 and 13	0.15 and 1.60
5	34.6	34.7	6	8	F	1976	180	0.34
6	30.9	36.4	9	10	M	1900	73	0.40
7	31.3	34.2	7	8	F	1550	35	0.59
8	31.7	32.8	6	9	M	1710	96	0.34
9	35	35.1	7	8	F	2350	21	4.25

Sub: Subject, GA: Gestational age, PMA: Postmenstrual age, F: Female, M: Male, QS/AS epoch ratio: the ratio of the number of QS (quiet sleep) to AS (active sleep) epochs in each subject’s recorded data. * This subject has been recorded twice on different days.

**Table 2:** List of the features (i.e., modalities) extracted from the NIRS signals accompanied by a description for each feature.

Feature	Description
O2Hb	Filtered O2Hb was obtained using a moving average filter of 1 s and down-sampled to 1 Hz.
HHb	Filtered HHb was obtained using a moving average filter of 1 s and down-sampled to 1 Hz.
TSI	Filtered TSI by using a moving average filter of 1 s and down-sampled to 1 Hz.
HR	Extracted HR from NIRS and interpolated to 1 Hz.
RR	Extracted RR from NIRS and interpolated to 1 Hz.
Moving standard deviation	Normalized standard deviation on O2Hb in sliding windows of 1 s overlapping by 50% and interpolated to 1 Hz.
Moving IQR	Normalized IQR on O2Hb in sliding windows of 1 s overlapping by 50% and interpolated to 1 Hz.
Moving correlation	Pearson’s correlation between O2Hb and HHb in sliding windows of 1 s overlapping by 50% and interpolated to 1 Hz.

**Table 3:** Optimized hyperparameters for benchmark classifiers.

Classifier	Hyperparameters
KNN ^1^	{‘Distance weight’: Squared inverse; {‘Number of Neighbors’ = 20}; {‘NS method: Exhaustive};
NB ^2^	{‘Kernel’: Normal − ‘Width’ = 0.05}
SVM ^3^	{‘Kernel’: Linear}; {‘Box constraints’ = 0.1}; {‘Kernel scale’ = 0.5};
RF ^4^	{‘n_estimators’: 100}; {‘max_depth’: 20}; {‘min_samples_split’: 2};
AdaB ^5^	{’Learning rate’= 0.5}; {‘Minimum Leaf Size’ = 8}; {‘Max Splits’ = 20}
XGB ^6^	{’Learning rate’= 0.2};{‘Max depth = 3}

^1^ K-Nearest Neighbors. ^2^ Naive Bayes. ^3^ Support Vector Machine. ^4^ Random Forest. ^5^ AdaBoost. ^6^ XGBoost.

**Table 4:** Quantitative metrics for assessing the performance of the algorithms used for extracting the physiological information, i.e., HR and RR, from NIRS, averaged across all measurements.

Physiological Information	ME ^1^ (BPM ^2^ or EPM ^3^)	RMSE ^4^ (BPM or EPM)	LoA ^5^ (BPM or EPM)	Pearson’s r (%) (*p* < 0.01)
HR	−1.4 ± 0.2	3.4 ± 0.2	6.0 ± 0.3	93.9 ± 4.2
RR	−2.1 ± 0.5	6.1 ± 0.2	11.1 ± 0.5	89.3 ± 6.6

^1^ Mean of error. ^2^ Beats per minute. ^3^ Events (i.e., breaths) per minute. ^4^ Root mean square error. ^5^ Bland–Altman limits of agreement.

**Table 5:** The average and standard deviation of quantitative metrics employed to assess classifier performance using the data pooling cross-validation approach, calculated across all measurements.

Classifier	Accuracy (%)	Balanced Accuracy (%)	F1-Score (%)	Kappa (%)	AUC-ROC (%)
CNN	88 ± 12	94 ± 7	91 ± 9	95 ± 5	96 ± 4
SVM ^1^	74 ± 4	68 ± 4	53 ± 7	35 ± 9	70 ± 6
AdaB ^2^	83 ± 4	77 ± 5	66 ± 9	54 ± 11	80 ± 6
KNN ^3^	82 ± 5	83 ± 6	71 ± 9	59 ± 12	84 ± 5
NB ^4^	63 ± 5	67 ± 5	51 ± 6	26 ± 8	68 ± 6
RF ^5^	88 ± 5	79 ± 6	72 ± 10	65 ± 12	85 ± 4
XGB ^6^	86 ± 3	79 ± 5	71 ± 8	62 ± 9	87 ± 3

^1^ Support Vector Machine. ^2^ AdaBoost. ^3^ K-Nearest Neighbors. ^4^ Naive Bayes. ^5^ Random Forest. ^6^ XGBoost.

**Table 6:** Average and standard deviation of quantitative metrics employed to assess classifier performance using the leave-measurement-out cross-validation approach, calculated across all measurements.

Classifier	Accuracy (%)	Balanced Accuracy (%)	F1-Score (%)	Kappa (%)	AUC-ROC (%)
CNN	78 ± 6	72 ± 6	59 ± 10	44 ± 14	75 ± 6
SVM ^1^	72 ± 5	69 ± 7	53 ± 11	35 ± 14	70 ± 5
AdaB ^2^	72 ± 2	61 ± 10	39 ± 22	22 ± 21	65 ± 8
KNN ^3^	69 ± 9	63 ± 8	46 ± 10	25 ± 16	66 ± 9
NB ^4^	68 ± 11	70 ± 8	55 ± 6	34 ± 14	68 ± 10
RF ^5^	78 ± 5	67 ± 11	45 ± 23	35 ± 21	75 ± 6
XGB ^6^	78 ± 5	69 ± 10	50 ± 21	38 ± 21	76 ± 5

^1^ Support Vector Machine. ^2^ AdaBoost. ^3^ K-Nearest Neighbors. ^4^ Naive Bayes. ^5^ Random Forest. ^6^ XGBoost.

**Table 7:** Quantitative metrics were employed to assess the performance of the proposed CNN classifier in each subject group, obtained using the leave-measurement-out cross-validation approach.

Group No.	Accuracy (%)	Balanced Accuracy (%)	F1-Score (%)	Kappa (%)	AUC-ROC (%)
1	86	81	74	65	88
2	76	72	51	36	75
3	70	63	48	27	72
4	84	75	66	56	86
5	73	70	56	37	74

## Data Availability

The raw data supporting the conclusions of this article will be made available by the authors on request. The videos that were used for sleep scoring of the subjects included in this study will not be made available because of the privacy of these subjects.

## Supplementary Materials

The following supporting information can be downloaded at: https://www.mdpi.com/article/10.3390/s24217004/s1, Figure S1: Labelled (AS and QS) average values of each feature in the pooled dataset; Table S1: Ranges of hyperparameter values for Grid search.
