# Supplementary material for: Near-Infrared Spectroscopy for Neonatal Sleep Classification
Source: Sensors (Basel). 2024 Oct 31;24(21):7004. doi: 10.3390/s24217004 (PMC11548375; doi:10.3390/s24217004)
Supplement: Supplementary file 1 [file sensors-24-07004-s001.zip › sensors-3245812-supplementary.pdf]

Table S1. Ranges of hyperparameter values for Grid search:

Algorithm	Hyperparameters	Value ranges and symbol
KNN	Distance weight	['uniform', 'distance', 'squared inverse']
	Number of Neighbors	[1, 5, 10, 15, 20, 25, 30]
	NS method	['auto', 'ball_tree', 'kd_tree', 'brute']
	Break ties	['smallest', 'largest', 'random']
NB	Kernel	['normal', 'epanechnikov', 'triangular']
	Width	[0.01, 0.05, 0.1, 0.2, 0.5]
SVM	Kernel	['linear', 'poly', 'rbf', 'sigmoid']
	Box constraints (C)	[0.01, 0.1, 1, 10, 100]
	Solver	['SMO', 'L-BFGS']
	Kernel scale	[0.1, 0.5, 1, 2, 5]
	Decision function shape	['ovo', 'ovr']
RF	n_estimators	[50, 100, 200, 300, 500]
	max_depth	[None, 10, 20, 30, 40, 50]
	min_samples_split	[2, 5, 10, 20]
	criterion	['gini', 'entropy']
AdaB	Number of learning cycles	[50, 100, 200, 300, 400, 500]
	Learning rate	[0.01, 0.1, 0.5, 0.8, 1.0]
	Minimum Leaf Size	[1, 2, 4, 8, 16]
	Max Splits	[2, 10, 20, 50, 100]
XGB	Learning rate	[0.01, 0.1, 0.2, 0.3, 0.5]
	Min split loss	[0, 0.1, 0.2, 0.5, 1]
	Max depth	[3, 4, 5, 6, 7, 8]

Figure S1 compares the values of each feature over 723 epochs:

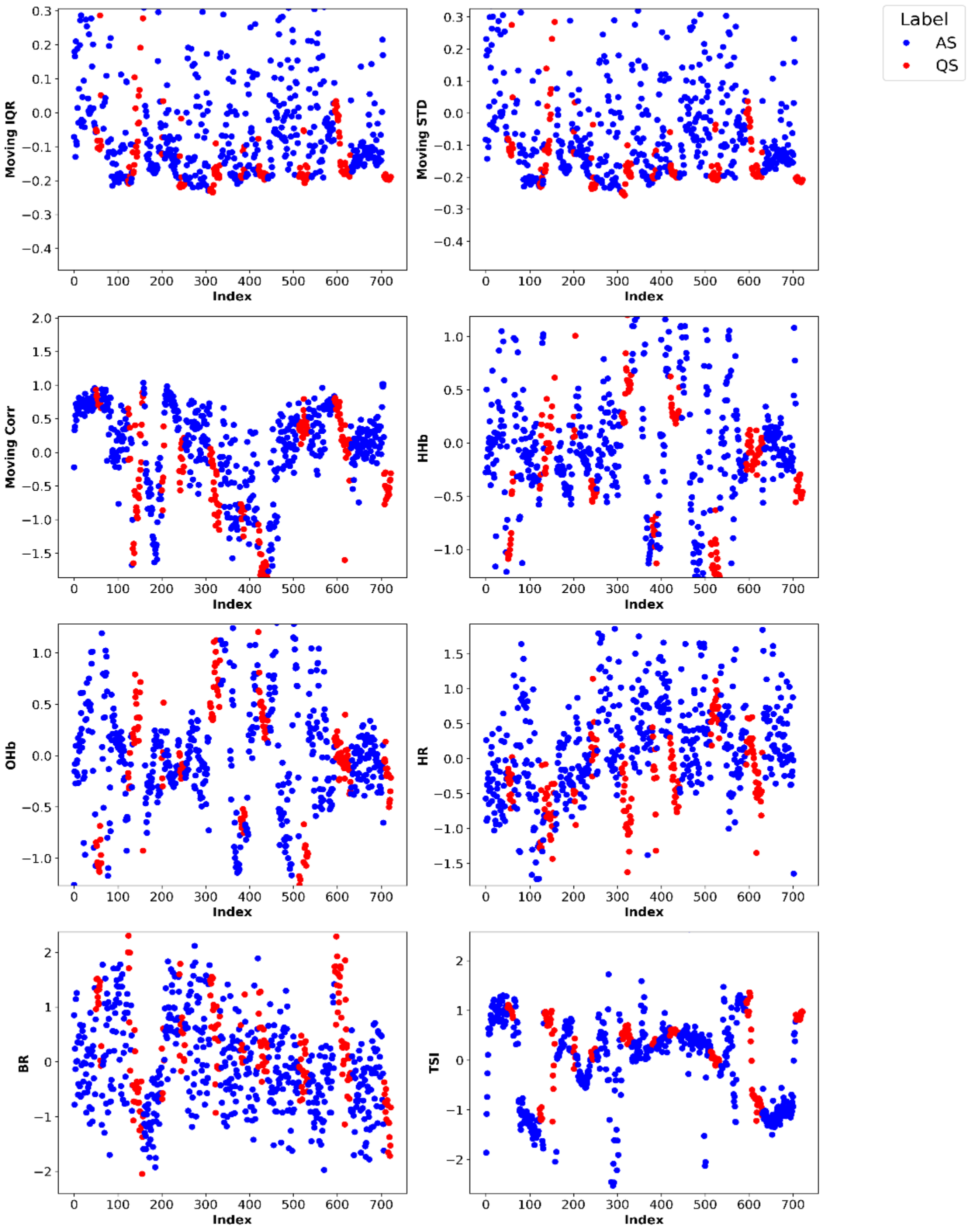

Figure S1. Average values of each feature over 723 epochs. “Moving Corr” and “Moving STD” stand for moving correlation and moving standard deviation, respectively. The index is the number of epochs in the pooled dataset.
